# Structural basis of ligand specificity and channel activation in an insect gustatory receptor

**DOI:** 10.1016/j.celrep.2024.114035

**Published:** 2024-04-03

**Authors:** Heather M. Frank, Sanket Walujkar, Richard M. Walsh, Willem J. Laursen, Douglas L. Theobald, Paul A. Garrity, Rachelle Gaudet

**Affiliations:** 1 Department of Molecular and Cellular Biology, Harvard University, 52 Oxford Street, Cambridge, MA 02138, USA; 2 The Harvard Cryo-EM Center for Structural Biology, Harvard Medical School, Boston, MA 02115, USA; 3 Department of Biological Chemistry and Molecular Pharmacology, Blavatnik Institute, Harvard Medical School, Boston, MA 02115, USA; 4 Department of Biochemistry, Brandeis University, Waltham, MA 02453, USA; 5 Department of Biology and Volen Center for Complex Systems, Brandeis University, Waltham, MA 02453, USA; 6 These authors contributed equally; 7 Lead contact

## Abstract

Gustatory receptors (GRs) are critical for insect chemosensation and are potential targets for controlling pests and disease vectors, making their structural investigation a vital step toward such applications. We present structures of *Bombyx mori* Gr9 (BmGr9), a fructose-gated cation channel, in agonist-free and fructose-bound states. BmGr9 forms a tetramer similar to distantly related insect odorant receptors (ORs). Upon fructose binding, BmGr9’s channel gate opens through helix S7b movements. In contrast to ORs, BmGr9’s ligand-binding pocket, shaped by a kinked helix S4 and a shorter extracellular S3-S4 loop, is larger and solvent accessible in both agonist-free and fructose-bound states. Also, unlike ORs, fructose binding by BmGr9 involves helix S5 and a pocket lined with aromatic and polar residues. Structure-based sequence alignments reveal distinct patterns of ligand-binding pocket residue conservation in GR subfamilies associated with different ligand classes. These data provide insight into the molecular basis of GR ligand specificity and function.

## In brief

Insect gustatory receptors are ligand-gated cation channels critical for chemosensation and constitute targets for controlling pests and disease vectors. Frank et al. use cryo-EM to determine *Bombyx mori* Gr9’s structure, showing its tetrameric organization and ligand-binding pocket composition, and the movements associated with ligand-gated ion-pore opening.

## Graphical abstract

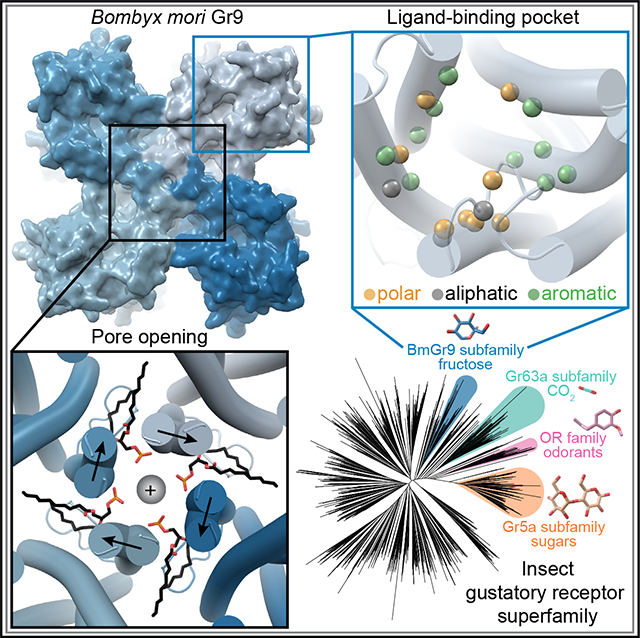

## INTRODUCTION

Animals rely on chemosensory systems to adjust their behavior and physiology in response to changing external conditions and internal states. Chemical detection often relies on the cell-specific expression of members of large families of membrane-spanning chemoreceptors.^1^ Given their central role in transduction, determining the three-dimensional structures of chemoreceptors is important to understand the molecular mechanisms, including ligand recognition and selectivity, that govern chemosensory system function.

Insects impact ecology and human health,^2,3^ with many providing vital ecosystem services like pollination^4^ and some acting as disease vectors^5^ or agricultural pests.^6^ Insect chemoreceptors influence feeding, reproduction, and other vital behaviors.^1,7^ Therefore, knowledge of insect chemoreceptor structures and mechanisms can influence the development and use of agents for conservation and control efforts.

Among arthropods, including insects, gustatory receptors (GRs) are important mediators of chemosensation,^8^ with 60 GR genes in *Drosophila melanogaster*,^8^ 72 in the dengue vector mosquito *Aedes aegypti*,^9^ and 65 in the silkworm *Bombyx mori*.^10^ Many GRs are expressed in gustatory neurons and regulate feeding.^1^ For example, in *D. melanogaster*, eight GRs contribute to sweet compound detection.^11^ GRs can also act in olfactory neurons, with disease-spreading mosquitoes using GR-mediated carbon dioxide (CO_2_) detection to help locate human hosts.^12^ GRs are also used to sense internal stimuli. In *D. melanogaster*, Gr43a monitors internal fructose levels to control satiety^13^ and egg production.^14^ Thus, GRs detect diverse chemical stimuli to control behavior and physiology.

GRs mediate chemosensation by forming ligand-gated cation channels.^15–17^ GRs belong to the seven-transmembrane ion channel (7TMIC) superfamily, a large group of proteins that share transmembrane topologies but little sequence identity.^18,19^ Insect odorant receptors (ORs) also belong to the 7TMIC superfamily.^8,18^ ORs diverged from within the GR family >400 million years ago and now form another large chemoreceptor family (e.g., *D. melanogaster* encodes 60 ORs) primarily involved in olfaction.^8^ ORs share limited sequence identity with GRs (<15%) but also function as ligand-gated cation channels.^20,21^ In winged insects, individual ORs assemble with a common OR coreceptor (ORCO) to form functional ion channels.^22^ While GRs lack an analogous coreceptor, many also function in a combinatorial fashion.^23^ However, some GRs do form functional homomeric channels, including *D. melanogaster* Gr43a and its *Bombyx mori* ortholog (Bm)Gr9, which are selectively activated by fructose.^16^ As BmGr9 is one of the few GRs known to form functional channels with native-like properties in heterologous expression systems,^16^ we chose BmGr9 as an initial GR family member for structural determination.

Among the ORs, there are structures of the fig wasp *Apocrypta bakeri* (Ab)Orco^24^ and the jumping bristletail *Machilis hrabei* (Mh) Or5, a homomeric OR that does not require an ORCO and is activated by a broad panel of odorants.^25^ While GRs and ORs are related, they are highly divergent, and they recognize chemically distinct agonists (from water soluble for many GRs to hydrophobic for many ORs). The similarity of GR and OR protein folds and the extent to which GRs and ORs exhibit unique structural features that support their distinct functions are not well known.

Here, we present structures of BmGr9 in both the closed agonist-free state and an open state in the presence of D-fructose. BmGr9 forms a homotetramer with a quadrivial pore architecture, and fructose binding results in ligand-binding pocket contraction and the replacement of hydrophobic by hydrophilic residues at the extracellular gate of the ion pore. A set of four ordered phospholipids lie horizontally in the plane of the membrane with their head groups interacting with conserved polar groups on the pore-lining transmembrane helix S7b and penetrating into the ion pore through intersubunit fenestrations. Finally, we generate structure-based sequence alignments and uncover distinct conservation patterns in the ligand-binding pocket in GR subfamilies associated with distinct ligand classes. Together, these findings provide a high-resolution view of a GR, identify specific functions for conserved residues in long-established GR sequence motifs, and provide a starting point for more comprehensive analyses of this large and divergent sensory receptor family.

## RESULTS

### Overall structure of an insect GR

**Figure 1.**
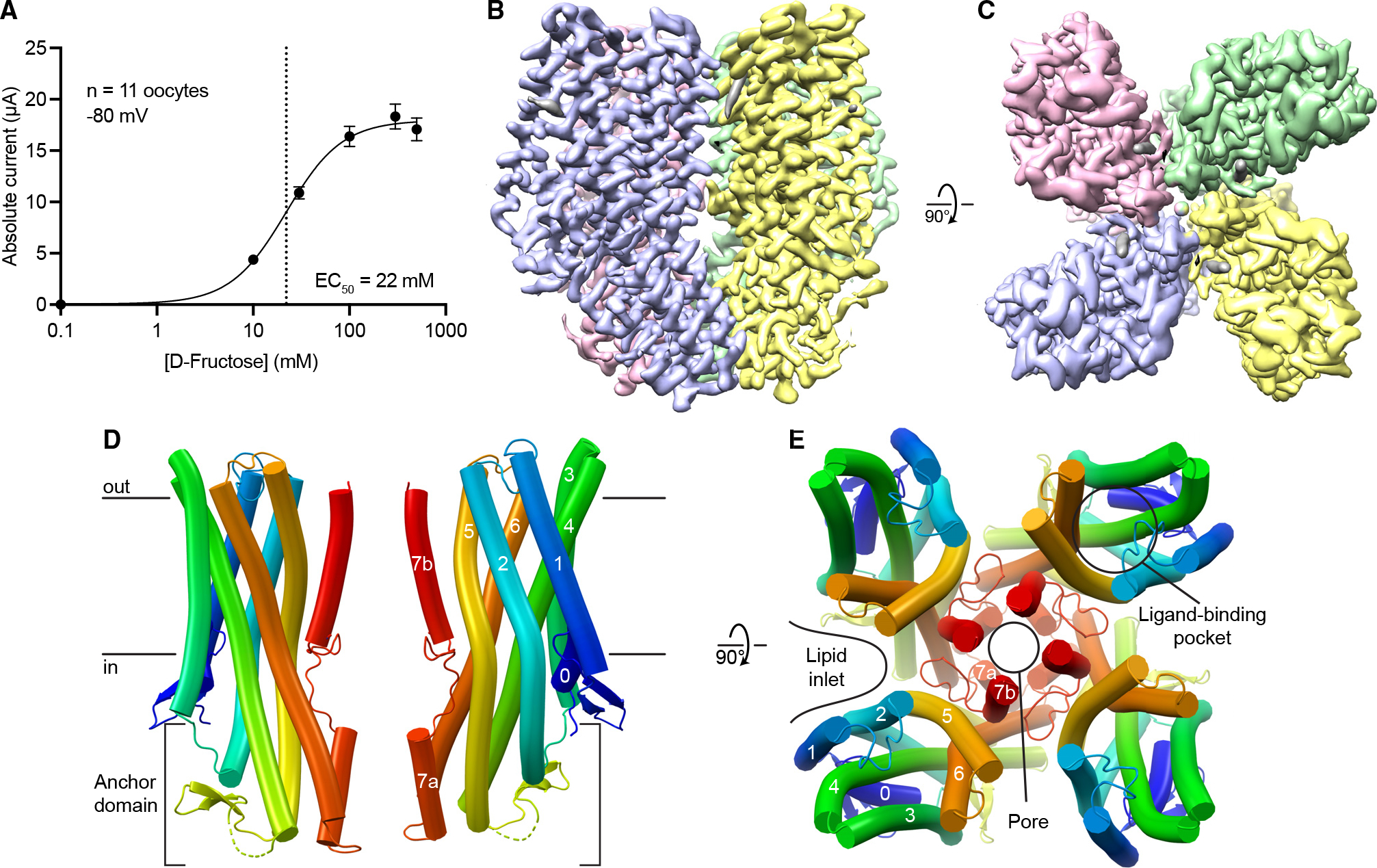
Cryo-EM structure of a tetrameric insect gustatory receptor, BmGR9. (A) D-Fructose dose response of Twin-Strep-tagged BmGR9 expressed in *Xenopus* oocytes. Currents measured at −80 mV (n = 11; means ± SEM; EC_50_ = 22 mM). (B) (B and C) Cryo-EM map of agonist-free BmGr9 (resolution 2.85 Å; contour level 0.24, carved around the protein) viewed from the membrane plane (B) and extracellular side (C). Each subunit is colored differently. (D) Cartoon representation of two opposing BmGr9 subunits viewed from the membrane plane. Black horizontal lines indicate the membrane boundaries. The helices of one subunit and the anchor domain are labeled. (E) BmGr9 tetramer viewed from the extracellular side. The pore and fructose-binding pocket and the helices of one subunit are labeled, as is one of the intersubunit lipid inlets. See also Figures S1–S5.

To determine the BmGr9 structure, we produced and purified N-terminally Twin-Strep-tagged BmGr9 using HEK293 cells. When expressed in *Xenopus* oocytes, this construct was activated by d-fructose, but not by D-glucose, with an EC_50_ of 22 mM (Figures 1A and S1A–S1D), consistent with the published EC_50_ and specificity of wild-type BmGr9.^16^ We produced detergent-solubilized BmGr9 (Figures S1 E–S1G) and used single-particle cryoelectron microscopy (cryo-EM) to obtain a 2.85-Å map (Figures 1B, 1C, S2, and S3; Table 1), which we used to build a structural model of BmGr9 (Figures 1D and 1E). BmGr9 purified in the presence of d-fructose (Figures S1F and S1H) yielded a 3.98-Å map, which we used to model the fructose-bound conformation (Figures S4 and S5; Table 1). The root-mean-square deviation (RMSD) between individual subunits of agonist-free and fructose-bound BmGr9 is 1.5 Å. We first describe the structural features of BmGr9 using the higher-resolution agonist-free structure and then compared the fructose-bound and agonist-free structures to define the fructose-mediated structural changes.

As anticipated from its homology to ORs, each BmGr9 subunit contains an N-terminal intracellular S0 helix and seven transmembrane helices (S1 to S7), with S7 broken into S7a and S7b (Figure 1D). BmGr9 is a C4-symmetric tetramer with the ion pore on the central 4-fold axis (Figure 1E). The BmGr9 structure encompasses most of the protein, except for a disordered intracellular S4-S5 loop (residues 232–269), and the N and C termini (15 and 5 residues, respectively). Helices S1 and S3 only span the thickness of the lipid bilayer, while S2, S4, S5, S6, and S7a extend into the intracellular space to form an anchor domain similar to that observed in AbOrco and MhOr5 (Figure 1D).^24,25^ Helix S7b contributes to the pore, and helices S0–S6 expand outward from the central pore to form the ligand-binding pocket (Figure 1E). The main intersubunit contacts are around the transmembrane pore and the intracellular anchor domain (Figures 1D and 1E), burying ~2,200 Å^2^ of surface area per subunit with 965 Å^2^ from the pore region and 1,235 Å^2^ from the anchor. The four anchor domains thus form a tight intracellular bundle, whereas deep lipid inlets largely isolate each subunit in the membrane plane (Figure 1E).

### BmGr9 and MhOr5 have distinct structural features

**Figure 2.**
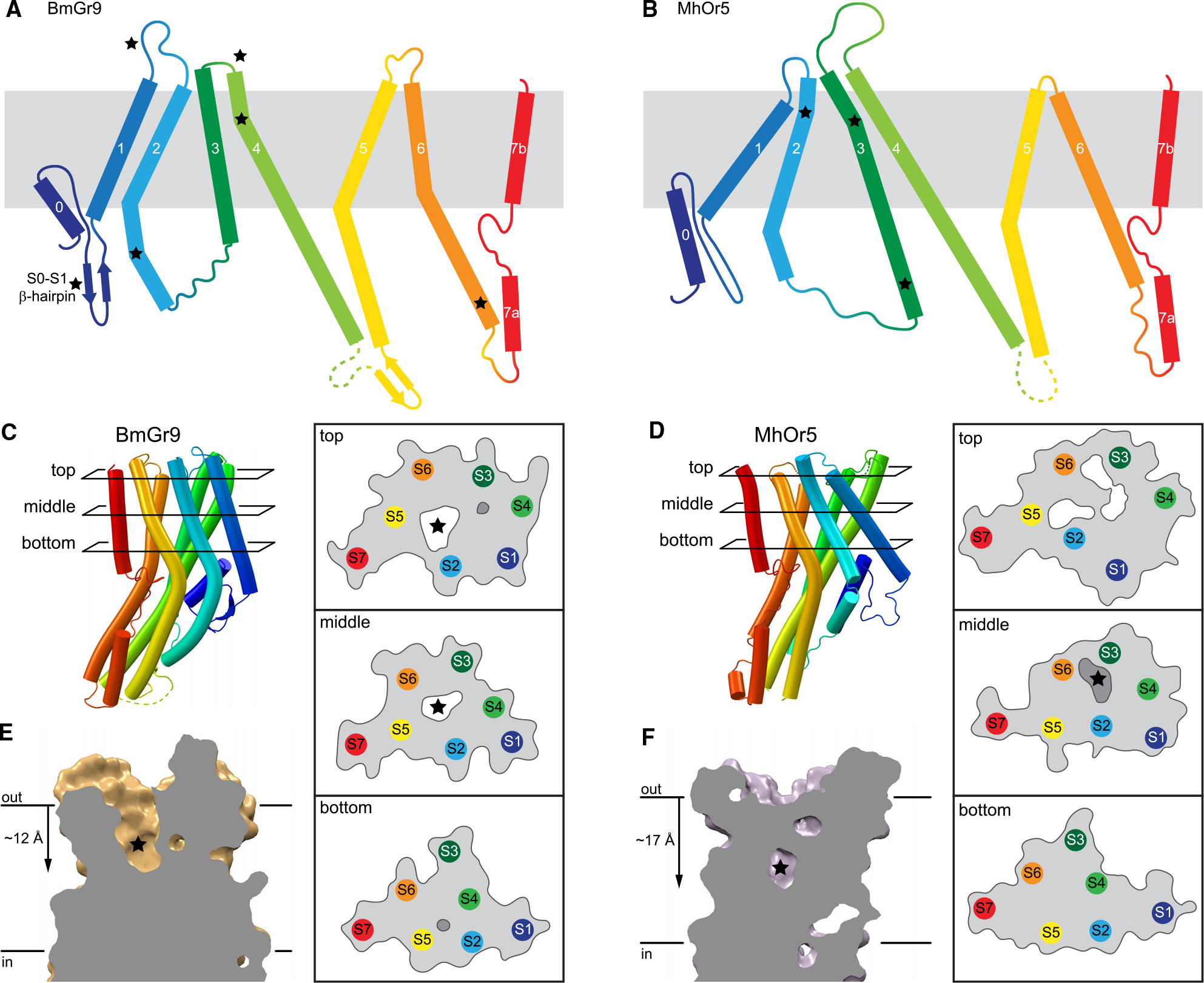
BmGr9 and MhOr5 have distinct structural features. (A and B) Topology diagrams of BmGr9 (A) and MhOr5 (B). Several features distinct between BmGr9 and MhOr5—loop and helix lengths, kinks in helices—are starred. (C and D) Helix packing arrangements and protein surface (gray line) and internal cavities (white, accessible to the extracellular solvent; darker gray, solvent occluded) of BmGr9 (C) and MhOr5 (D) at top, middle, and bottom cross-sections across the membrane plane as indicated on the subunit on the left. Stars mark the BmGr9 fructose-binding pocket (C) and MhOr5 eugenol-binding pocket (D). (E and F) Vertical slices through the ligand-binding pocket in the transmembrane domain of one subunit of BmGr9 (E) and MhOr5 (F). Stars mark ligand positions. Distances from the extracellular membrane boundary to the pocket bottom are on the left. See also Figures S6 and S7.

Both AbOrco and MhOr5 assemble as tetramers with a central ion pathway formed by the C-terminal S7b.^24,25^ Each MhOr5 subunit has a ligand-binding site in the middle of the membrane bilayer plane that appears inaccessible to aqueous solvent, consistent with its selectivity for hydrophobic ligands.^25^ Based on superpositions of individual agonist-free subunits, BmGr9 is more distant from AbOrco and MhOr5 than they are from each other (RMSDs = 5.1, 4.2, and 3.4 Å for the BmGr9-AbOrco, BmGr9-MhOr5, and AbOrco-MhOr5 pairs, respectively; full RMSD matrix in Table S1). The secondary structure elements of BmGr9 are well defined in our cryo-EM maps (Figure S6); several elements differ in length and orientation compared to MhOr5 (Figures 2A and 2B).

The most prominent structural differences between BmGr9 and MhOr5 are starred in Figures 2A and 2B. They include that (1) BmGr9 has a β-hairpin between the S0 and S1 helices, which neatly tucks under S0 (Figure 2C); (2) the S1-S2 loop is longer in BmGr9 than in MhOr5 (10 versus 4 residues) and the S3-S4 loop—which closes access to the eugenol-binding pocket in MhOr5—is much shorter in BmGr9 than in MhOr5 (1 versus 28 residues); (3) BmGr9’s S2 extends further into the cytosol (7 residues longer than in MhOr5) and is more curved, with two kinks that allow it to pack against the rest of the anchor domain; (4) S6 is longer in BmGr9 (45 versus 34 residues), with a sharp kink at the intracellular membrane boundary; and (5) BmGr9’s S4 has another sharp kink near its extracellular tip.

The combination of these five structural differences generates two global differences between BmGr9 and MhOr5. First, the different orientations and positions of the S1–S6 helices within the membrane plane (Figures 2C and 2D) and the lengths of the extracellular loops (Figures 2A and 2B) generate very different ligand-binding pockets (Figures 2E and 2F). BmGr9 has a ~12-Å deep solvent-accessible pocket that totals ~600 Å^3^ in volume, whereas MhOr5 has a much smaller pocket (~100 Å^3^) that is deeper in the membrane plane and occluded from extracellular solvent by its longer S3-S4 loop. Furthermore, S5 lines the fructose-binding pocket in BmGr9, whereas MhOr5’s S5 does not contact the eugenol-binding pocket (top and middle cross-sections in Figures 2C and 2D). Second, the presence of the S0-S1 β-hairpin and the longer S2 helix in BmG9 provide more extensive structural connections between the intracellular anchor domain and the ligand-binding pocket formed by helices S1–S6 in the transmembrane domain.

To assess whether the structural features discussed above are linked to specific sequence signatures, we created separate family wide sequence alignments of 1,854 GRs and 3,885 ORs (excluding ORCOs) from insects using a structure-based approach leveraging the AlphaFold Protein Structure Database^26^ (see STAR Methods for details). The coverage of the two alignments is excellent, particularly at alignment positions corresponding to secondary structure elements (Figure S7A). From the aligned GRs, we extracted a subalignment of 74 Gr43a subfamily members containing BmGr9 and the other experimentally validated fructose receptor orthologs. We also used a published alignment of 176 ORCO sequences.^24^

Sequence covariation among the 1,854 aligned GRs, with 28 strongly evolutionarily coupled residue pairs (>90% confidence; Figures S7B and S7C), supports structural elements observed in both GRs and ORs and the idea that several of the structural features distinct to BmGr9 in comparison to MhOr5 are conserved in GRs. One pair cross-links the two S0-S1 β-hairpin strands, suggesting that this β-hairpin is conserved in GRs. Another pair is in the transmembrane region, corresponding to the buried salt bridge between E200 and R361 just below the base of the ligand-binding pocket. Intriguingly, this salt bridge is conserved in MhOr5 (D220–R387) but not in AbOrco, suggesting that it is best conserved in subunits that naturally bind ligands. The twenty-six remaining pairs are intrasubunit contacts within the anchor domain, and several link the intracellular extension of the S2 helix to S4 and S5. This suggests that the extended S2 (when compared to MhOr5; Figures 2A and 2B), and its packing against S4 and S5 to expand the anchor domain, is a conserved feature in GRs. This S2-anchor domain connection could provide additional leverage for allosteric communications between the ligand-binding pocket and other regions of the tetrameric ion channels, including the ion pore and its gate.

Covariation analysis of the 3,885 aligned OR sequences reveals 82 evolutionarily coupled pairs, most in the anchor domain like in GRs. However, unlike in GRs, 21 pairs connect the ligand-binding pocket-lining helices and 5 pairs connect either S5 or S6 to the pore-forming S7b (Figures S7D and S7E). The presence of coupled pairs surrounding the ligand-binding pocket is consistent with a conserved structural scaffold in ORs, which generally respond to similar small hydrophobic volatile compounds. In contrast, the comparative lack of strong couplings in the transmembrane region of GRs likely reflects their broader structural divergence to accommodate their vast functional diversity, which we explore further below.

### Lipid head groups penetrate the ion pore through intersubunit fenestrations

**Figure 3.**
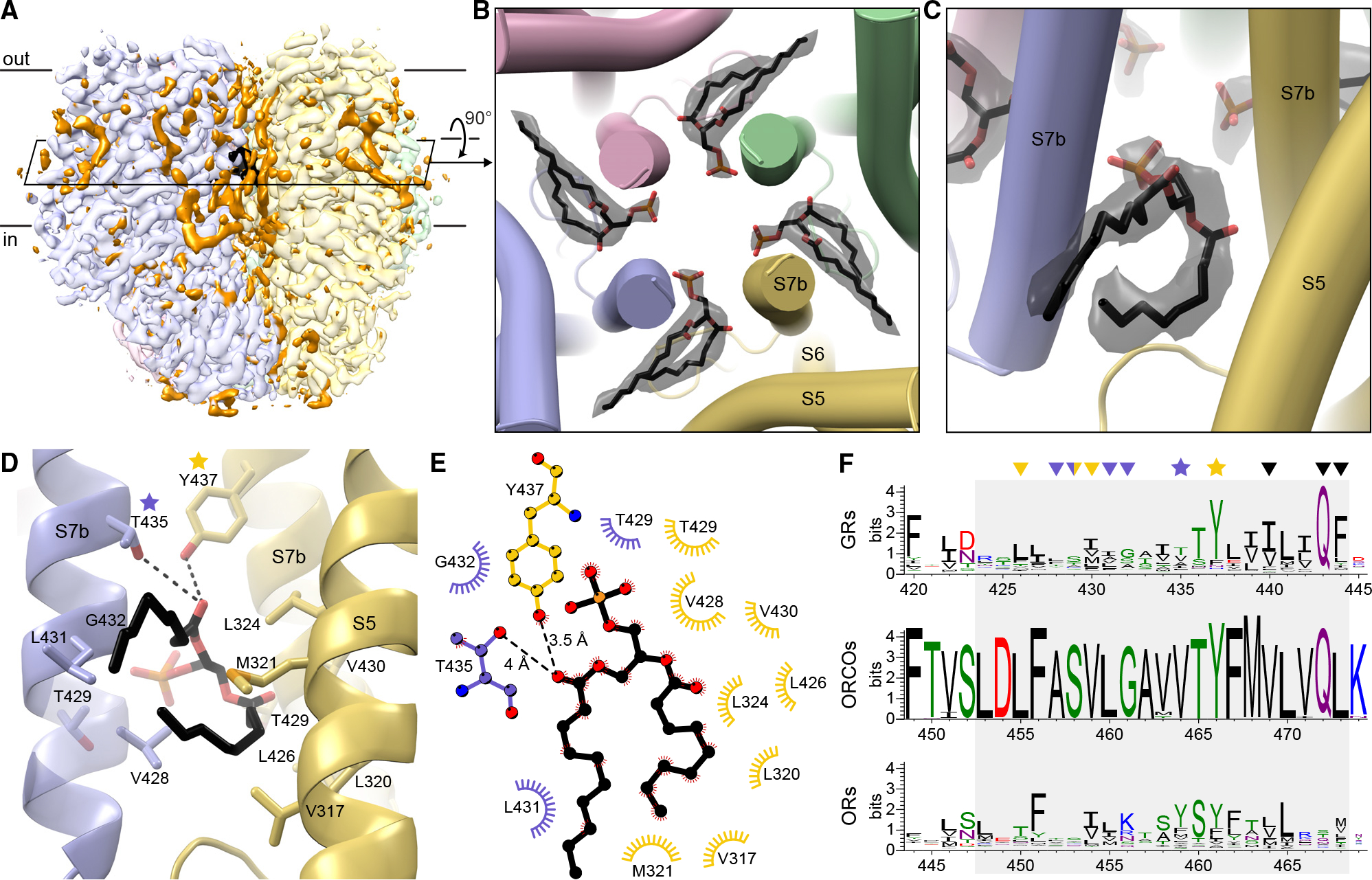
Lipid head groups penetrate the BmGr9 pore through intersubunit fenestrations. (A) Cryo-EM map of agonist-free BmGr9 including non-protein densities (contour level 0.254). Each subunit is in a different pastel color; non-protein densities are orange except for those of the pore-penetrating lipids, which are black. The black parallelogram marks the cross-section in (B). (B) BmGr9 cross-section viewed from extracellular side highlighting densities (black transparent surface) for lipids with head groups penetrating into the pore. Modeled lipids are in sticks (carbon in black, oxygen in red, phosphorus in orange). Only the phosphate of head groups is modeled, as the rest are disordered and lack density. (C) View from the membrane plane of a pore-penetrating lipid and its corresponding density highlighting its bidentate shape. (D) View from membrane plane with side chains within 4.2 Å of the pore-penetrating lipid as sticks. The head group contacts S7b helices from two subunits (blue and yellow, respectively), whereas a tail makes hydrophobic contacts with the yellow S5. One turn of the blue S7b is transparent so that T429 is visible. Two conserved polar residues are starred. (E) Schematic of the lipid-protein interactions colored as in (D). (F) Sequence logos of S7b positions (gray box) from alignments of insect GRs, ORCOs, and insect ORs (green, polar amino acids [G, S, T, Y, C, Q, N]; blue, basic [K, R, H]; red, acidic [D, E]; and black, hydrophobic [A, V, L, I, P, W, F, M]). Residues interacting with the pore-penetrating lipids are marked with stars (polar) or arrowheads (aliphatic) colored according to (D) and (E). Black arrowheads mark pore-gating residues. See also Figures S8 and S9.

The cryo-EM map of agonist-free BmGr9 contains strong density features that do not correspond to protein, especially in the intersubunit lipid inlets (Figure 3A). We assigned several features to ordered phospholipids rather than detergent based on their well-resolved bidentate shapes. Their head groups are unmodeled beyond the phosphate due to missing density. Because no lipids were added during protein purification, these lipids likely remained bound to BmGr9 through sample preparation.

Four ordered lipids (one per subunit) project their head group from within the hydrophobic region of the bilayer into the aqueous ion pore, sealing intersubunit fenestrations in the pore walls (Figures 3B and 3C). Each lipid enters the pore between adjacent S7b helices at the level of the inner leaflet. Interestingly, while associated lipids were not reported for AbOrco and MhOr5,^24,25^ analogous fenestrations capable of accommodating lipids are observed in both structures (Figures S8A and S8B). Furthermore, bidentate densities consistent with such a pore-penetrating lipid are observed in our fructose-bound BmGr9 map and in the eugenol-bound and N,N-diethyl-*meta*-toluamide (DEET)-bound MhOr5 maps (Figures S8C–S8E).^25^ The position of these pore-penetrating lipids suggests that they could contribute to the structural stability of the pore, influence ion conduction and selectivity, or both.

In BmGr9, T435 and Y437 form polar contacts with the pore-penetrating lipid head group (Figures 3D and 3E). Tyrosine is highly conserved at the Y437 position across GRs (80.7%; Figure 3F) and ORCOs (99.4%; position 466 in AbOrco) and corresponds to the second residue in the GR-family signature motif TYhhhhhQF^27^ (where h is any hydrophobic residue). Tyrosine is also common at the corresponding position in ORs (52.9%; position 461 in MhOr5). Small polar residues are common at the T435 position in GRs (34.6% S, T, or N), while tyrosine is common in ORs (51.1%), preserving the potential to hydrogen bond with lipid head groups. The pore-penetrating lipid hydrophobic tails interact with non-polar side chains from S5 and S7b. As expected from their membrane-embedded positions, hydrophobic residues are enriched at each of these positions in GRs, ORCOs, and ORs (Figures 3D–3F).

In summary, three observations suggest that the presence of pore-penetrating lipids is a conserved feature across both GRs and ORs: all available structures have intersubunit fenestrations between S7b helices, lipid-shaped densities are present in similar positions in cryo-EM maps of both BmGr9 and MhOr5, and the general properties of the lipid-interacting residues are conserved in sequence alignments of GRs and ORs.

To investigate the role of these pore-penetrating lipids, we performed 200-ns molecular dynamics simulations of three agonist-free, bilayer-embedded BmGr9 systems:without pore-penetrating lipids or with pore-penetrating lipids containing either phosphatidylcholine or phosphatidylethanolamine head groups. Phosphatidylcholine and phosphatidylethanolamine are the most common lipids in HEK293 cells^28^ and insect cells,^28–30^ respectively. All systems were well equilibrated with a backbone RMSD ~2 Å (Figure S9A). The BmGr9 pore readily accommodates either phosphatidylcholine or phosphatidylethanolamine head groups, and the lipid tails explore the interstitial lipid inlet space (Figures S9B and S9C). Comparing the pore hydration across the three systems, the absence of pore-penetrating lipids leads to water leakage into the intersubunit fenestrations (Figures S9D–S9F), supporting a role for these lipids in the ion pathway’s structural integrity.

### Pore gate opening involves an interaction network conserved between GRs and ORs

**Figure 4.**
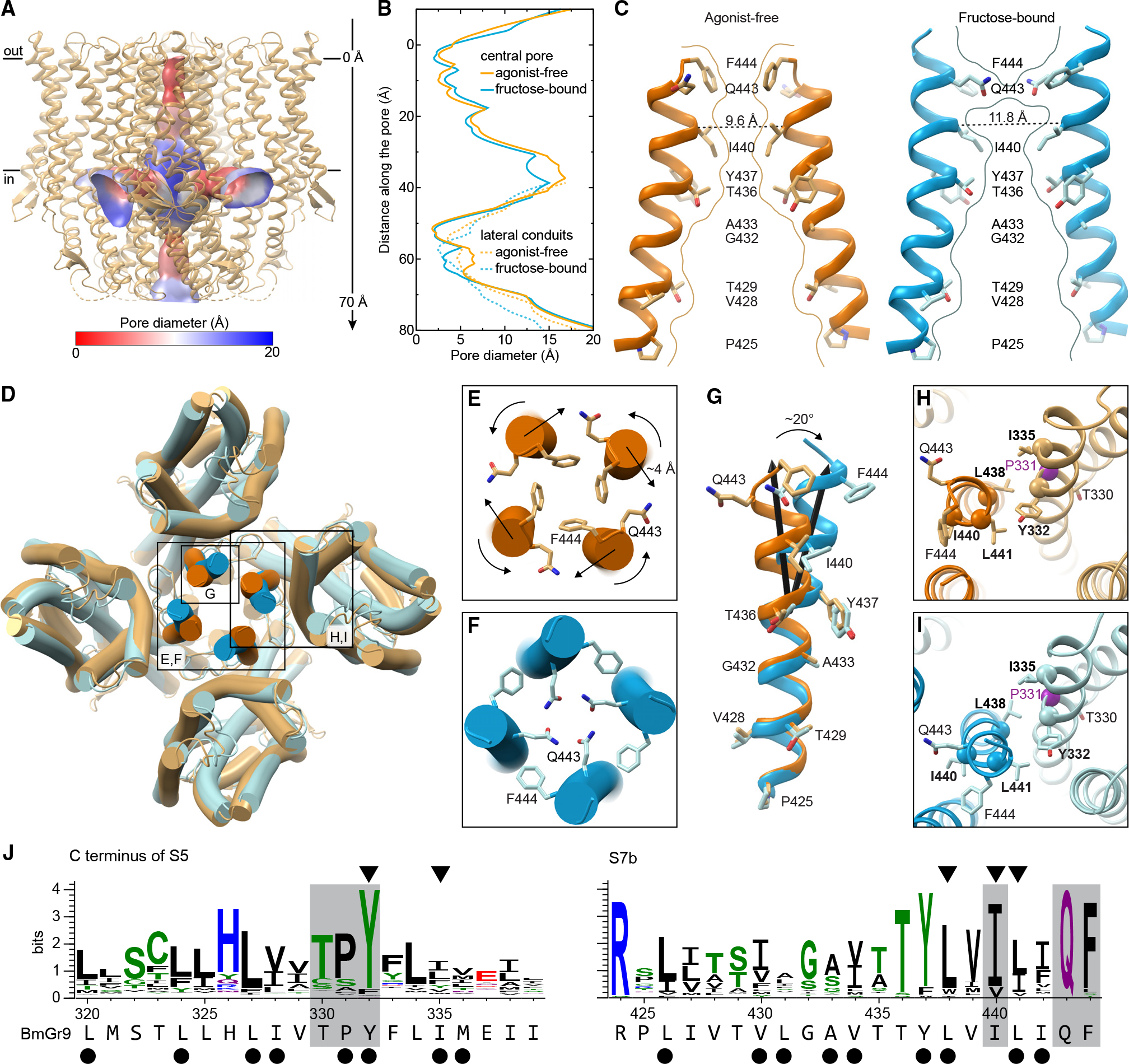
Fructose binding induces pore opening in BmGr9 through concerted motions of a conserved network of residues. (A) Agonist-free BmGr9 with its quadrivial pore as a surface colored according to its diameter. The vertical axis for (B) is indicated on the right. (B) Diameter of the central ion pathway (solid lines) and lateral conduits (dashed lines) of agonist-free (orange) or fructose-bound (blue) BmGr9. The y axis shows the distance from the outer membrane boundary toward the cytosol for the central pore or the distance along the lateral conduit. (C) Two opposing S7b helices with the pore-lining side chains in sticks for agonist-free (left) and fructose-bound (right) BmGr9. Curved lines mark the pore surface at the central vertical cross-section. Dashed lines indicate Cα-to-Cα distances between opposing hydrophobic gate I440 residues. (D) Superposition of agonist-free (orange) and fructose-bound (blue) BmGr9. Inset (E)–(I) are indicated. (E and F) Extracellular views of the agonist-free (E) and fructose-bound (F) BmGr9 pore with conserved gating Q443 and F444 side chains in sticks. Arrows indicate twisting motions of gating residues and displacement of the S7b C termini. (G) S7b viewed from the central pore of the superimposed agonist-free (orange) and fructose-bound (blue) BmGr9 tetramer structures. Pore-lining side chains are in sticks and labeled. Black arrows mark the central axes of the S7b top half, highlighting the 20° kink and displacement upon fructose binding. (H and I) S5-S7b interactions in agonist-free (H) and fructose-bound (I) BmGr9. Purple Cα sphere marks P331. Alanine mutations of residues in bold and marked by the other Cα spheres increase basal currents.^31^ Pore-gating Q443 and F444 and the conserved, pocket-facing T330 are in sticks for reference. (J) Sequence logos of the C-terminal half of S5 and of S7b for the Gr43a subfamily. The BmGr9 sequence is below. Black dots mark hydrophobic S5-S7b interface residues. Arrowheads mark locations where alanine mutations increased basal currents.^31^ Gray highlights mark the conserved TPY motif in S5 and pore-gating residues in S7b. See also Figure S10.

The BmGr9 porehas the same quadrivial architecture as AbOrco^24^ and MhOr5^25^ with a singular extracellular path down the tetramer’s center that opens into a large vestibule in the middle of the membrane plane and then diverges into four lateral conduits formed between subunits (Figures 4A and S10A–S10D). The density for pore helix S7b and its side chains is excellent in both the agonist-free and fructose-bound BmGr9 maps (Figure S6). In agonist-free BmGr9, starting from the extracellular face, the pore begins with a closed double-layer hydrophobic gate formed by F444 and I440 at the S7b C terminus (Figures 4A–4C). Like L473 in AbOrco and V468 in MhOr5 (Figure S10E), the four F444 side chains protrude into the channel lumen to close the ion pathway down to a 2.5-Å diameter, creating a hydrophobic plug (Figures 4A–4C). Below the hydrophobic gate, T429 and T436 form two rings of hydroxyls that can stabilize cations (Figure 4C). AbOrco and MhOr5 also have hydroxyls in the same positions (Figure S10E). T436 is highly conserved across insect GRs—it is the first residue of the TYhhhhhQF motif. T429 is less conserved although predominantly polar or charged (71%, 100%, and 74% across GRs, ORCOs, and ORs, respectively; Figure S10F).

Immediately below F444 near the extracellular pore opening, Q443 points away from the pore in agonist-free BmGr9 but rotates into the ion pathway in fructose-bound BmGr9 (Figures 4C–4F). The Q443 side chains thus create a hydrophilic ring more suitable for cation conduction in the fructose-bound structure, although the pore diameter is largely unchanged (Figure 4B). In addition to its twisting along the helix axis, S7b moves out from the tetramer center by ~4 Å. T436, near the pore-penetrating lipid head group, and acts as a fulcrum, such that only the portion of S7b C-terminal to T436 moves (Figure 4G). As a result, the pore widens the most at the other hydrophobic constriction: the Cα-to-Cα distance between opposing I440 residues increases from 9.6 to 11.8 Å and the pore diameter from 2.5 to 3.2 Å (Figures 4B and 4C).

Q443 and F444 are highly conserved in GRs and ORCOs; they are the last two residues of the TYhhhhhQF motif (Figure S10F). In BmGr9, the motif adheres to the GR consensus: 436-TYLVILIQF-444. This motif is notably less conserved in ORs, most of which form heteromers with ORCO^8,20^; however, this glutamine is conserved in homotetrameric MhOr5, which is from a basal insect species that lacks an ORCO ortholog.^32^ This Q467 in MhOr5 also swings into the pore upon agonist-induced channel opening, and mutations to alanine or arginine eliminated agonist-activated calcium influx, suggesting that this rearrangement is critical for receptor function.^25^

Mutations of hydrophobic gate residues I440 and F444 in BmGr9 also support a role for both in gating.^31^ I440A and I440Q increased the resting current and decreased the responsiveness to fructose compared to wild type, suggesting that the hydrophobic gate was disrupted, allowing cation flux without agonist binding.^31^ F444A increased the EC_50_ for fructose and reduced fructose-induced currents, suggesting a change in open-closed equilibrium or conductance.^31^

Pore helix S7b is tightly packed with—and its movements coupled to—helix S5, which connects directly to the binding pocket in BmGr9 (Figures 4H–4J). BmGr9 residues L441 (S7b) and Y332 (S5) correspond to MhOr5 residues L465 and Y362, respectively, two highly conserved and evolutionarily coupled hydrophobic residues in ORs that also move in concert between the agonist-free and eugenol-bound MhOr5 structures.^25,33^ The Y362A mutation in MhOr5 impaired eugenol activation but Y362F did not, supporting the importance of the aromatic group at this position.^33^ BmGr9 residue Y332 is highly conserved in GRs, while L441 is conserved as aliphatic (Figure 3F). Alanine mutations to Y332, L441, or two other conserved aliphatic residues at the S7b-S5 interface—L438 and I335—increased BmGr9 resting currents,^31^ suggesting that tight packing of S7b and S5 is required for proper ligand gating. Although mutations at corresponding residues in BmGr9 and MhOr5 yield different functional impairments, both impact receptor function, supporting the importance of the S5-S7b interaction in GRs and ORs.

The S5-S7b interaction connects the pore to the ligand-binding pocket, suggesting that small fructose-induced movements in S5 could gate the pore. Y332 is part of a highly conserved TPY motif in the Gr43a subfamily (Figure 4J). T330 faces the pocket and likely interacts with the fructose (see also below). P331 breaks the backbone hydrogen-bonding pattern on S5 and thus could reduce the energy barrier to local rearrangements and facilitate allosteric communication between the ligand-binding pocket and the pore.

### Fructose-binding pocket is lined with polar and aromatic residues

**Figure 5.**
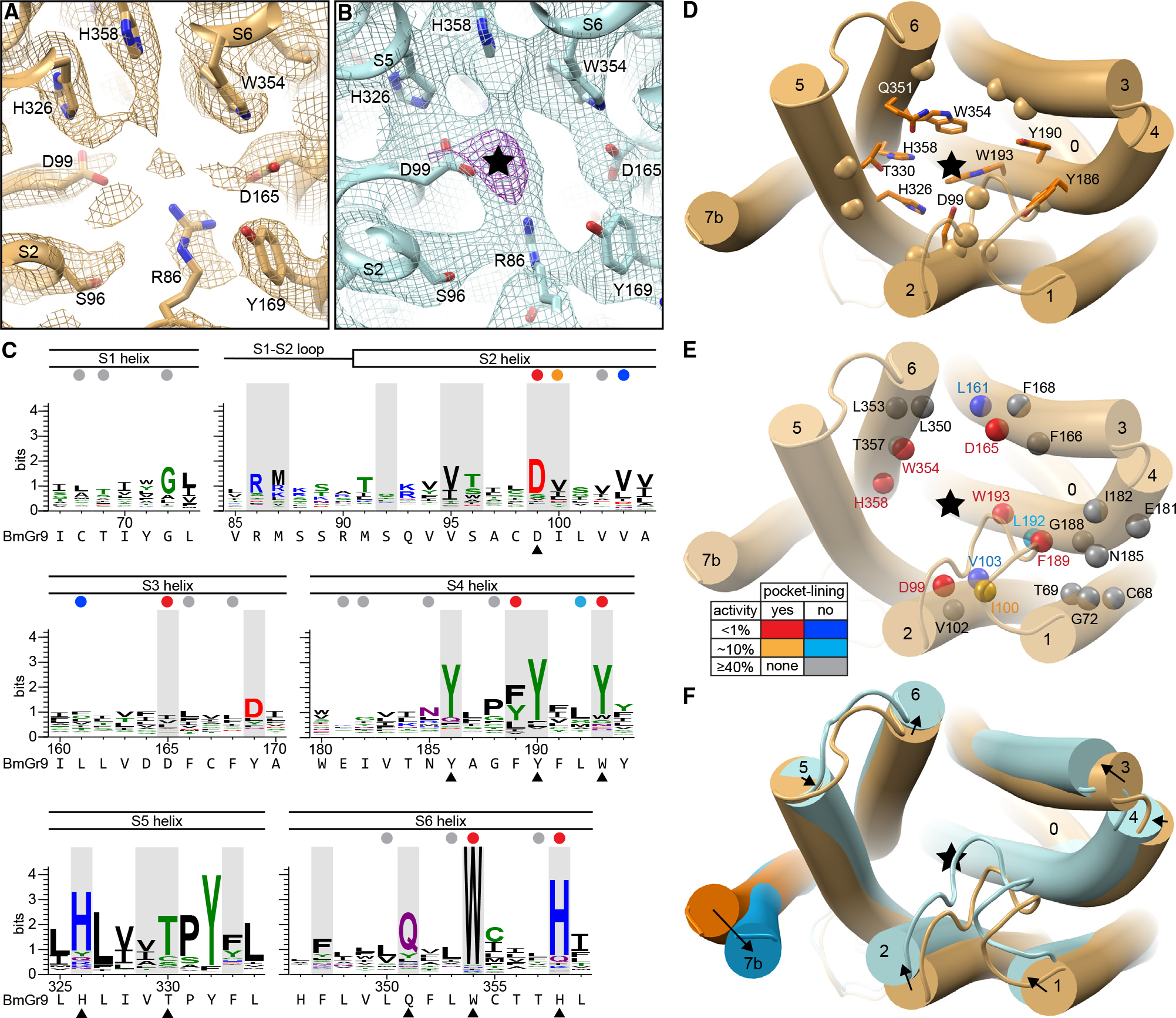
BmGr9 ligand-binding pocket and its fructose-induced conformational changes. (A) Cryo-EM density around the ligand-binding site for agonist-free BmGr9 contoured at 3.5 σ. Nearby side chains are in sticks and labeled. (B) Density around the ligand-binding site for fructose-bound BmGr9 contoured at 4.5 σ (cyan). The black star marks strong >10 σ density (magenta) not observed in the agonist-free map in (A). (C) Sequence logos from the Gr43a subfamily covering the ligand-binding pocket regions; corresponding secondary structure elements indicated above and BmGr9 sequence below. The 21 pocket-lining positions are shaded gray. Black arrowheads mark conserved pocket-facing residues highlighted in (D). Colored circles mark positions assessed by alanine substitutions in BmGr9^31^ and highlighted in (E) (red, pocket-lining and <1% activity compared to wild-type BmGr9; orange, pocket-lining and ~10% activity; blue, outside pocket and <1% activity; cyan, outside pocket and ~10% activity; gray, outside pocket and ≥40% activity). (D–F) Cartoon views of one BmGr9 subunit from the extracellular side with black stars marking the fructose position. (D) Pocket-lining residues are in sticks for highly conserved residues or marked by Cα spheres for others (corresponding to black arrowheads and gray highlights in C, respectively). (E) Positions previously assessed by alanine substitutions^31^ are shown as spheres. The side-chain orientation and mutation effect are represented by colors as described in (C) and in the table. (F) Superimposed subunits of agonist-free (orange) and fructose-bound (blue) BmGr9 with black arrows highlighting the helix movements from the agonist-free state to the fructose-bound state. See also Figure S11.

In contrast to the solvent-inaccessible eugenol-binding cavity in MhOr5,^25^ BmGr9 has a deep solvent-accessible cavity at the analogous location, with its opening near the center of each subunit on the extracellular face (Figures 2, S11A, and S11B). In each subunit, the extracellular termini of helices S0–S6 splay out, with helices S2–S5 forming the walls of this deep pocket. At the bottom of this pocket, the fructose-bound map contains additional density when compared to the agonist-free map (Figures 5A and 5B). While the map resolution is not high enough to resolve the fructose binding pose, the location is consistent with published mutational analysis.^31^ Furthermore, computational docking suggests that a fructose molecule is readily accommodated in this pocket: when docking a β-d-fructopyranose (the most abundant form of d-fructose in solution^34^), the most energetically favored positions are just adjacent to the observed density (Figure S11C). Upon refinement of the protein model and docked fructose against the cryoEM map, the fructose roughly fits inside the density (Figure S11C). We are therefore confident in assigning the bottom of the pocket as the fructose-binding site and discuss it as such below, although we refrain from describing specific fructose-protein interactions.

Nine of the 21 residues lining the fructose-binding pocket are highly conserved in the Gr43a subfamily (>66% identity; Figures 5C and 5D). As described above, the TPY motif on S5 connects the pocket to the pore movements (Figures 4H and 5D). S5 residue T330, along with H326, faces the binding pocket. Both are highly conserved and near other highly conserved polar and aromatic residues at the pocket bottom (S2 residue D99; S4 residues Y186, Y190, and W193 [68.6% Y and 80% aromatic]; and S6 residues Q351, W354, and H358). Of the 21 pocket-lining residues, 7 have been previously investigated by mutagenesis.^31^ Of those, conserved residues D99, W193, W354, and H358, as well as D165 and F189 (70% conserved as aromatic), are all essential for BmGr9 function, as mutating each residue individually to alanine dramatically reduced or eliminated fructose-activated channel function^31^ (Figure 5E). Two additional residues (of 16 tested) adjacent to but not lining the pocket, V103 and L161 (81.7% and 58.0% aliphatic, respectively), were also important for the fructose response (Figure 5E); they may structurally enable allosteric communication between the ligand-binding site and the pore gate. These mutagenesis data are in better agreement with our structure than with the homology model used to design the mutations, providing strong support to our fructose-binding site assignment. The high sequence conservation in the pocket (9 of the 45 most conserved residues in the Gr43a subfamily), particularly at its bottom, suggests that maintaining aromatic and hydrophilic side chains is important for ligand binding and is an important first step to convert fructose binding to pore opening.

In both a superposition of the agonist-free and fructose-bound BmGr9 subunit structures (Figure 5F) and an alignment-free distance difference matrix (Figure S11D), we observe agonist-induced movements of helices S2–S6 that reshape the binding pocket (Figures 5F, S11A, and S11B). S2—with its highly conserved D99 residue closest to the fructose density—moves the most, and most helices move inward toward the central axis of the subunit (Figure 5F). This coordinated movement of the helices could convey changes caused by fructose binding to the gates of the ion pore.

### Ligand-binding pocket comparison across the GR family

**Figure 6.**
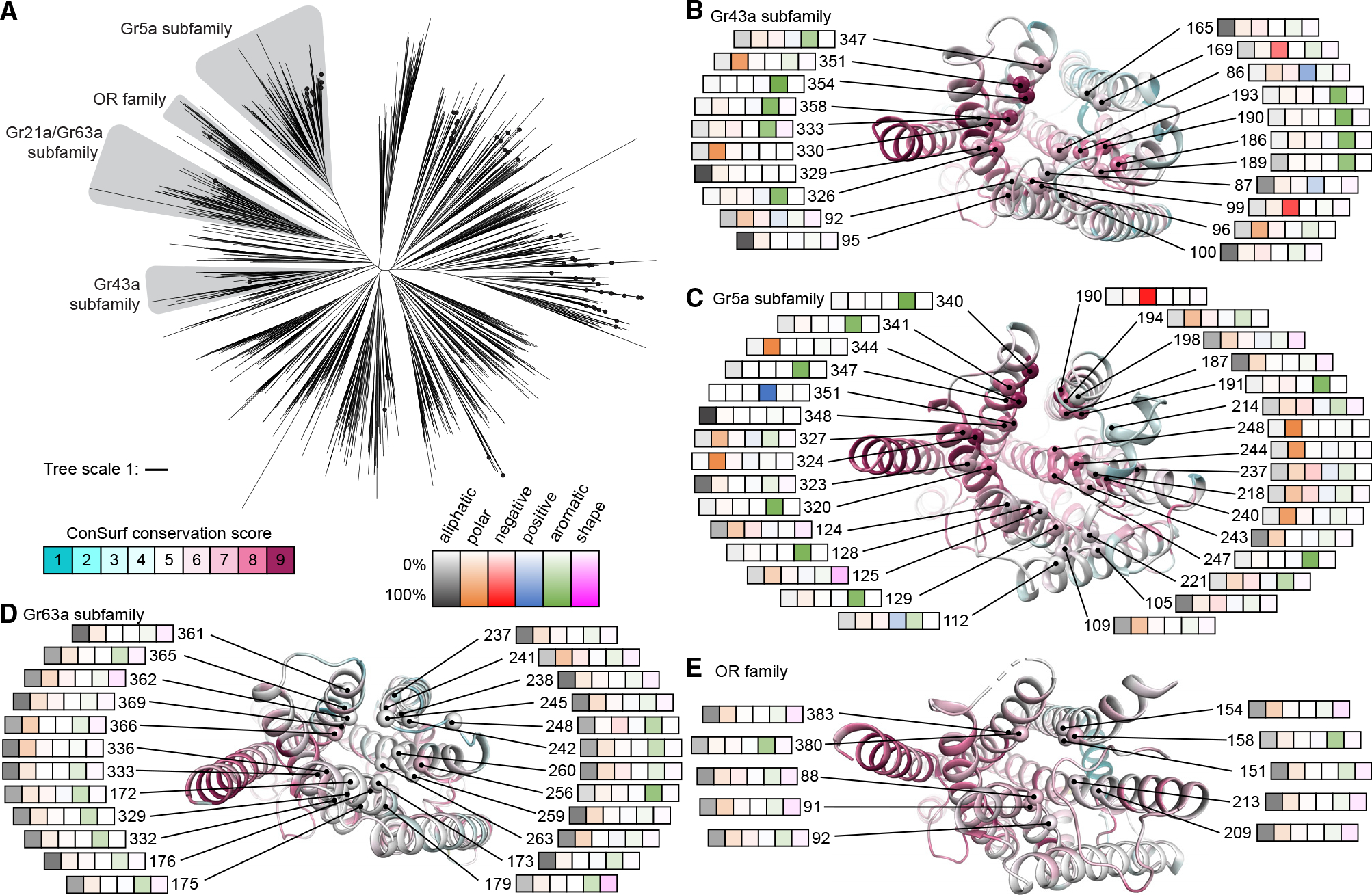
Ligand pocket comparison across GR family. (A) Phylogenetic tree of insect GRs with subfamilies of interest highlighted gray. The tree includes a clade of 41 ORs. Black dots represent *Drosophila* GRs. (B–E) Subunits of representative subfamily members viewed from the extracellular side. Cartoons are colored by ConSurf^32^ score based on the corresponding GR subfamily sequence alignment. Cα spheres mark predicted pocket-lining residues labeled with their residue numbers in the reference protein and heatmaps capturing amino acid-type frequencies at that position in the corresponding alignment (left to right are aliphatic [Ala, Cys, Leu, Met, Val; black], polar [Asn, Gln, Ser, Thr; orange], negatively charged [Asp, Glu; red], positively charged [Arg, Lys; blue], aromatic [His, Phe, Trp, Tyr; green], or shape-determining [Gly, Pro; pink] residues). The following (sub)families and corresponding subunits are illustrated: (B) Gr43a subfamily on agonist-free BmGr9, (C) Gr5a subfamily on the Gr5a model, (D) Gr63a subfamily on the Gr63a model, and (E) OR family on agonist-free MhOr5. See also Figures S12 and S15.

Using the BmGr9 structure as a reference, we explored the potential of bioinformatic approaches to inform structure-function relationships among GRs more broadly. To assess evolutionary relationships among GRs, we inferred a phylogenetic tree from our sequence alignment of 1,854 GRs and 41 ORs (Figures 6A and S12). As chemical detection is a primary function of these receptors, we focused our analysis on the putative ligand-binding pockets for four subfamilies containing members with relatively well-established chemosensory functions: the Gr5a and Gr43a subfamilies, which mediate sugar sensing; the Gr63a subfamily, which mediates CO_2_ sensing; and the ORs, which respond to volatile organic compounds (Figures 6A and S13).

We leveraged experimentally determined structures of MhOR5 (OR) and BmGr9 (Gr43a subfamily) and AlphaFold predictions for Gr5a or Gr63a subfamily members. We assessed the accuracy of AlphaFold2 predictions of GRs and ORs by comparing the monomeric models of BmGr9 and AbOrco from the AlphaFold database^26^ to their experimentally determined agonist-free structures (the database has no MhOr5 model). While neither structure was in the AlphaFold2 training set, both models correspond well to the respective structures (RMSDs of 0.9 Å for BmGr9 and 0.3 Å for AbOrco). We thus used the AlphaFold models of *D. melanogaster* Gr5a and Gr63a to identify likely ligand-binding pockets and pocket-lining residues (Figure S14). Using ConSurf,^32^ we mapped sequence conservation across 251 Gr5a and 107 Gr63a subfamily members to the corresponding representative AlphaFold models and 74 Gr43a subfamily members and 3,885 ORs to the structures of BmGr9 and MhOr5, respectively (Figures 6B–6E and S14).

While the Gr5a and Gr43a subfamilies are evolutionarily divergent (Figure 6A), both contain receptors involved in sugar detection, raising the possibility of similarities in their ligand-binding pockets. Indeed, despite their distinct evolutionary histories, their putative pockets are similarly enriched for aromatic and polar residues. Among Gr43a subfamily members, nine of 21 pocket-lining positions are most commonly occupied by aromatic residues, along with four by polar, two by acidic, one by basic, and five by aliphatic residues (Figure 6B). Among Gr5a subfamily members, 11 of the 31 pocket-lining positions are most commonly occupied by aromatic residues, 12 by polar, one by acidic, one by basic, and six by aliphatic residues (Figure 6C). Of the most highly conserved positions (ConSurf score ≥ 8), six are aromatic, two polar, and one negatively charged in the Gr43a subfamily (Figure 6B), while seven are aromatic, seven polar, two aliphatic, one positively, and one negatively charged in the Gr5a subfamily (Figure 6C). This abundance of aromatic, polar, and charged residues is consistent with the role of aromatic and hydrophilic residues in sugar binding in other proteins.^35,36^ As detailed below, it is not shared by Gr63a subfamily members or ORs.

Despite similarities in the chemical character of the binding pockets between the Gr43a and Gr5a subfamilies, their pocket sizes differ dramatically (Figures S14A, S14B, and S14E). The predicted Gr5a pocket is much larger than the observed BmGr9 pocket (~2,100 versus ~600 Å^3^; Figure S14E). The larger pocket in Gr5a arises from several key predicted structural differences: Gr5a’s longer S3-S4 loop forms a V-shape with two short helices that enable S3 and S4 to splay further from the subunit center to enlarge the pocket, while its shorter S1-S2 loop further widens the opening (Figure S15B). These shorter S1-S2 and longer S3-S4 loops are a distinct feature of Gr5a subfamily members compared to other GRs or to ORs (Figure S15A; *p* < 0.01 Steel-Dwass test). These larger pockets could accommodate a wider variety of sugars, consistent with the ability of gustatory neurons expressing Gr5a subfamily members to respond to polysaccharides as well as monosaccharides^11^ (Figure S14).

The Gr63a subfamily forms another divergent GR clade whose members can form CO_2_ sensors^1,12^ (Figure 6A). In contrast to the sugar receptors above, aliphatic residues predominate in the putative ligand-binding pockets of Gr63a subfamily members, with 20 of 24 pocket-lining positions mostly occupied by aliphatic residues and just three by aromatic and one by polar residues (Figure 6D). Similarly, for ORs, which are primarily involved in the detection of volatile organic compounds, 8 of 10 pocket-lining positions are most commonly occupied by aliphatic residues, with the remaining two by aromatic residues (Figure 6E). The pocket sizes of representatives of both of these subfamilies are substantially smaller than those of the sugar receptors (~150 Å^3^ for the Gr63a AlphaFold model and ~100 Å^3^ for MhOr5; Figures S14C–S14E). Thus, receptors implicated in detecting different classes of chemicals differ in both pocket size and chemical composition, consistent with key roles in determining ligand specificity.

## DISCUSSION

Our work reveals structural features that underlie the chemoreceptor activity of an insect GR. The BmGr9 homotetramer resembles a distantly related OR, MhOr5, including its quadrivial channel architecture. However, BmGr9 contains an additional S0-S1 β-hairpin and a longer S2 helix, which provide more extensive structural connections between the anchor domain and the transmembrane regions compared to MhOr5. BmGr9’s ligand-binding pocket also differs from MhOr5’s: the BmGr9 pocket is larger and involves additional receptor surfaces, is lined with aromatic and polar residues rather than hydrophobic residues, and is open to the extracellular milieu. These contrasting characteristics provide a structural basis for distinct functions with MhOr5 acting as a receptor for volatile, hydrophobic odorants like eugenol and BmGr9 as a receptor for fructose, a water-soluble carbohydrate. Sequence analyses indicate that these distinctive features are conserved across other ORs and Gr43a subfamily members. Fructose binding to BmGr9 elicits small movements of the S1–S6 helices that narrow the ligand-binding pocket. Movement of S5, which interacts with both the bound fructose and the S7b pore helix, potentially promotes the pore-opening motion of S7b.

Although GRs exhibit substantial sequence diversity, they share a characteristic sequence motif in the pore-forming helix S7b: TYhhhhhQF.^27^ In BmGr9, we associate a clear function with each of the four most highly conserved motif residues. The TY pair shapes the structure and hydrophilic character of the ion pathway: the four T436 side chains form a hydrophilic ring below the second hydrophobic gate—formed by the middle hydrophobic residue (I440) in the motif—while the Y437 side chains interact with pore-penetrating phospholipid head groups. The QF pair participates in gating. The four F444 side chains form a hydrophobic plug at the extracellular opening of the pore in the closed agonist-free state. Upon fructose binding, these phenylalanines swing out as the adjacent Q443 side chains swing into the pore, making the opening hydrophilic. Conservation of this motif across GRs suggests that helix S7b’s contributions to BmGr9 function are conserved in other GRs.

### Pore-penetrating lipids as a common feature of GRs and ORs

Based on the experimental structural data for BmGr9, AbOrco,^24^ and MhOr5,^25^ as well as the conservation of the TY sequence motif in GRs, ORs, and ORCOs, the pore-penetrating lipids bound to BmGr9 are likely conserved across the superfamily. All structures have pore fenestrations, and lipid-like density is present in both agonist-free and fructose-bound BmGr9 maps, suggesting that the lipids form part of a stable pore structure. Fenestrations opening the ion path to the membrane environment have been observed in other ion channel families, including ionotropic glutamate receptors, cysteine-loop receptors, voltage-gated potassium channels and sodium channels, and mechanosensitive two-pore domain potassium channels.^37–41^ In some channels, these fenestrations are transient, while in others, they are observed in both closed and open channel states. In many cases these fenestrations allow activators or blockers—including endogenous ligands—to enter the pore and have been implicated in the mechanisms of well-established drugs including local and general anesthetics.^41,42^ The fenestrations in insect GRs and ORs could therefore also represent opportunities for chemical modulation of their activity.

### Ligand-binding pockets of evolutionarily divergent sugar-sensing GRs share common features

The BmGr9 ligand-binding pocket differs significantly from a previously reported homology model, which was not AlphaFold based.^31^ Only 7 of 21 residues that participate in the pocket in our structures are shared with the earlier prediction.^31^ Observed contributions to the pocket from the S5 helix and the S1-S2 extracellular loop were not predicted in the earlier model, and the pocket-lining residues in S2, S3, S4, and S6 largely differ. Strikingly, six of the eight mutations from that study that dramatically reduced fructose-induced currents^31^ face the experimentally determined binding pocket. In contrast, of the 11 mutations with no effect,^31^ none face the pocket. These mutagenesis results suggest that residues distributed around the pocket participate in fructose binding and channel gating.

Our bioinformatics analyses indicate that the ligand-binding pockets of Gr5a subfamily members—a second set of GRs involved in sugar sensing—share characteristics with those of the Gr43a subfamily. As the two sugar-receptor subfamilies are otherwise evolutionary divergent, this likely reflects convergence. The larger pockets of Gr5a subfamily members also suggest a structural explanation for the responsiveness of gustatory neurons expressing Gr5a subfamily members to di- and poly- as well as monosaccharides,^11^ whereas BmGr9 and its orthologs like Gr43a are fructose specific. Finally, our bioinformatics analyses suggest that members of the Gr63a subfamily of CO_2_ receptors have highly hydrophobic binding pockets (Figure 6). This suggests that CO_2_ detection could involve hydrophobic interactions with pocket residues or the binding of a hydrophobic ligand or cofactor. Taken together, our work illustrates that the combination of experimental structures and structure-based sequence analyses can help delineate the evolution and function of large protein families like the insect GRs and ORs.

### Limitations of the study

The depth of our bioinformatic analysis is constrained by the paucity of functional information. Most attempts to study GRs in heterologous systems have failed to observe channel activity, and while genetic evidence suggests that many GRs form heteromers, which GRs hetero-oligomerize and which GRs participate in ligand binding or serve primarily structural roles remain to be established. A second limitation of our bioinformatic analyses is that while AlphaFold2 predictions agree well with the experimental structures of AbOrco and BmGr9, some predictions may not be as accurate. However, the presence of shared, and potentially convergent, features in the ligand-binding pockets of evolutionarily divergent sugar receptors, and their contrast with receptors for other classes of chemicals, supports the utility of bioinformatics approaches for capturing structural features on a scale not readily achieved by experimental determination alone.

### Note added in revision

While this work was in review, structures and mutational analyses of *D. melanogaster* Gr43a and Gr64a (a Gr5a family member), and of BmGr9, were published.^43,44^ Both studies largely corroborate our findings. All studies agree on the fructose-binding site and pore-opening mechanism of BmGr9 and its ortholog Gr43a. However, while both our and the other^44^ fructose-bound BmGr9 structures show Q443 lining the pore, this phenylalanine-to-glutamine gate switch was only observed in Gr43a-I418A structures, with the I418A mutation leading to increased basal currents (like I440A in BmGr9).^31,43^ The Gr43a and Gr64a maps show density assigned to pore-penetrating lipids, and molecular dynamic simulations of Gr43a with pore-penetrating phosphatidylcholine lipids yield results similar to ours.^43^ Moreover, the predictions from our Gr5a subfamily sequence analysis are confirmed by the Gr64a structure and functional analyses^43^: a larger and flatter ligand-binding pocket in Gr64a is buttressed by the long S3-S4 linker and accommodates disaccharide agonists like sucrose and maltose.

## STAR★METHODS

### RESOURCE AVAILABILITY

#### Lead contact

Further information and requests for resources and reagents should be directed to and will be fulfilled by the lead contact, Rachelle Gaudet (gaudet@mcb.harvard.edu).

#### Materials availability

Plasmids generated are available from the lead contact with a completed materials transfer agreement.

#### Data and code availability

- The cryo-EM maps have been deposited to the Electron Microscopy DataBank (EMDB) (accession numbers: EMD-43129 and EMD-43130, respectively, for the agonist-free and fructose-bound maps), and the refined coordinates to the Protein DataBank (PDB IDs: 8VC1 and 8VC2, respectively, for the agonist-free and fructose-bound BmGr9 structures). The GR family sequence alignment and phylogenetic tree, OR family sequence alignment, and molecular dynamics trajectories have been deposited in Dryad (https://doi.org/10.5061/dryad.cc2fqz6dp). All other data are available from the corresponding authors upon reasonable request.
- This paper does not report any original code.
- Any additional information required to reanalyze the data reported in this paper is available from the lead contact upon request.

### EXPERIMENTAL MODEL AND STUDY PARTICIPANT DETAILS

The *E. coli* DH5α strain, cultured in LB medium (Thermo Fisher Scientific) at 37°C, was used to amplify plasmids. Human embryonic kidney (HEK) 293F inducible GnTI- suspension cells (Thermo Fisher Scientific, A39242) were cultured in Expi293 expression medium (Thermo Fisher Scientific) at 37°C, supplied with 8% CO_2_. HEK293T cells were cultured in Dulbecco’s modified Eagle’s medium (DMEM, Corning) supplemented with 10% (v/v) fetal bovine serum (Corning), 1X GlutaMAX (Gibco), and 100 U/mL penicillin-streptomycin (Lonza) at 37°C and 5% CO_2_. *Xenopus laevis* oocytes (EcoCyte Bioscience) were cultured at 18°C in ND96 media containing (mM): 2 KCl, 96 NaCl, 2.0 MgCl_2_, 1.8 CaCl_2_, 5 HEPES-NaOH pH 7.4 supplemented with penicillin/streptomycin.

### METHOD DETAILS

#### Constructs

For protein expression and cryo-EM, the BmGr9 sequence (gift from Kazushige Touhara)^16^ was inserted into the pHR-CMV-TetO2_3C-Twin-Strep lentiviral expression plasmid with a IRES EmGFP reporter (Addgene ID: 113883) that was modified to introduce an N-terminal Twin-Strep tag (WSHPQFEKGGGSGGGSGGSAWSHPQFEK). For electrophysiology experiments, the same N-terminally tagged BmGr9 open reading frame was inserted into pOX (Addgene ID:3780) using Gibson assembly. For fluorescence-detection size exclusion chromatography (FSEC), BmGr9 was inserted into the pHR-CMV-TetO2_EmGFP lentiviral expression plasmid (Addgene ID:113892) modified to include an N-terminal EmGFP tag with a 3C protease cleavage site linker (AALEVLFQGPAAA) between EmGFP and BmGr9.

#### FSEC

HEK293T cells were seeded at 3×10^5^ cells in a 35-mm well plate, then transfected the next day with 3 μg of the EmGFP-BmGr9 expression plasmid using polyethylenimine (Polysciences). Cells were harvested 48 h post-transfection, washed with phosphate buffered saline (PBS; Corning), resuspended in 300 μL of solubilization buffer (PBS, 1 mM pepstatin A, 1 mM phenylmethylsulfonylfluoride (PMSF), 1 mM benzamidine, and 1% (w/v) n-dodecyl β-D-maltoside (DDM; Anatrace)), then rocked for 1.5 h at 4°C. This whole-cell lysate was cleared by centrifugation for 10 min at 18,000 g, then the insoluble material was pelleted at 92,000 g for 75 min at 4°C. The resulting supernatant (200 μL) was injected onto a Superose 6 10/300 GL column (Cytiva) equilibrated with FSEC buffer (20 mM TrisHCl pH 8.25, 150 mM NaCl, 1 mM DTT, 1 mM EDTA, 0.05% (w/v) DDM). The fluorescence of the eluate was measured using an FP-2020 fluorometer (Jasco) with an excitation wavelength of 450 nm, emission wavelength of 525 nm, gain of 1000, and bandwidth of 40 nm.

#### Stable polyclonal cell line for BmGr9 expression

BmGr9 was expressed using a lentivirus expression system to create a stable polyclonal cell line in the Expi293F inducible GnTI- cell line (Thermo Fisher Scientific) using published protocols.^45^ Briefly, lentiviral particles were produced by co-transfecting 1.8×10^7^ HEK293T cells seeded 24 h prior with 16 μg of the lentiviral expression plasmid described above, 16 μg psPAX2 packaging plasmid (Addgene ID: 12260), and 16 μg pMD2.G envelope plasmid (Addgene ID: 12259) using polyethylenimine (Polysciences). The lentivirus-containing supernatant was harvested after 3 days, then applied to 10×10^6^ Expi293F inducible GnTI- cells and incubated for 3 days to allow for genomic integration and establishment of a polyclonal stable cell line. Transduced cells were expanded and frozen in aliquots for long-term storage and use.

#### Protein expression and purification

The stably transduced polyclonal cell line was grown at 37°C with 8% carbon dioxide in Expi293 expression medium (Thermo Fisher Scientific) to 3×10^6^ cells/mL, then induced with 5 mg/mL doxycycline and 5 μM sodium butyrate for 72 h. Cells were collected by centrifugation at 600*g* for 10 min, washed with PBS, resuspended in lysis buffer (20 mM Tris pH 8.25, 150 mM NaCl, 1 μM pepstatin A, 1 mM PMSF, 1 mM benzamidine), and lysed using an Avestin Emulsiflex C5 with 4 passes at 5–15 kpsi. The lysate was cleared using a 15 min low-speed spin (9,700 g) and membranes were pelleted at 185,000 g for 2 h, flash frozen, and stored at −80°C. All purification steps were performed at 4°C. Thawed membranes from 2 L of culture were homogenized in 120 mL solubilization buffer (PBS, 1 μM pepstatin A, 1 mM PMSF, 1 mM benzamidine, and 2% (w/v) DDM) using a glass Potter-Elvehjem grinder, then rocked for 1.5 h at 4°C. Detergent-insoluble material was pelleted at 185,000 g for 40 min and the supernatant was loaded onto a 1-mL column of Strep-Tactin XT 4Flow resin (IBA Lifesciences) at 1 mL/min at 4°C. The resin was washed 5 × 2 column volumes (CV) of wash buffer (100 mM Tris pH 8.0, 150 mM NaCl, 1 mM EDTA, 0.05% (w/v) DDM, 1 mM DTT) then 6 × 0.5 CV of elution buffer (wash buffer with 50 mM biotin). To elute BmGr9, the column was rocked for 30 min with 3 CV of elution buffer and collected by displacing the column with 1 additional CV. The rocking elution was repeated 4–5 times until all protein was eluted. Elutions containing BmGr9 were combined and further purified using size exclusion chromatography (SEC) on a Superose 6 10/300 GL (Cytiva) equilibrated with SEC buffer (20 mM Tris-HCl pH 8.25, 150 mM NaCl, 1 mM DTT, 1 mM EDTA, 0.01% glyco-diosgenin (GDN; Anatrace)). For the fructose-bound structure the SEC buffer also contained 270 mM d-fructose. BmGr9-rich fractions were combined, concentrated to 3.2–3.3 mg/mL with a 100-kDa molecular weight cut-off centrifugal filter (Millipore), and used immediately for preparing cryo-EM grids.

#### Cryo-EM sample preparation

Concentrated BmGr9 protein (3 μL) was deposited onto 400 mesh Quantifoil Cu 1.2/1.3 grids that had been glow discharged in a PELCO easiGLOW (Ted Pella) at 0.39 mBar, 15 mA for 30 s. Agonist-free samples were vitrified in 100% liquid ethane using a Vitrobot Mark IV (Thermo Fisher Scientific), with a wait time of 0 s, blot time of 3 s, drain time of 0 s, and a blot force of 0 at 100% humidity. Fructose-bound samples were prepared with a blot time of 9 s.

#### Cryo-EM data collection and processing

Cryo-EM data were collected on a 300 kV Titan Krios G3i Microscope (Thermo Fisher Scientific) equipped with a K3 direct electron detector (Gatan) and a GIF quantum energy filter (20 eV) (Gatan) using counted mode at the Harvard Cryo-Electron Microscopy Center for Structural Biology at Harvard Medical School. Data were acquired utilizing image shift and real-time coma correction by beamtilt using the automated data collection software SerialEM^51^; nine holes were visited per stage position acquiring two movies per hole. Details of the data collection and dataset parameters are summarized in Table 1. Dose-fractionated images were gain normalized, aligned, dose-weighted and summed using MotionCor2.^52^ Contrast transfer function (CTF) and defocus value estimation were performed using CTFFIND4.^53^ Details of the data processing strategy are shown in Figure S2. In short, particle picking was carried out using crYOLO^54^ followed by 3D classification within Relion.^55^ The initial model for the agonist-free BmGr9 structure was generated *ab initio* and a low-pass filtered version of the agonist-free structure was used as the fructose-bound starting model. For the fructose-bound sample, a second round of 3D classification was performed to further improve the particle set (Figure S4). The selected particles were then subjected to Bayesian polishing following 3D refinement with C4 symmetry imposed. The data then underwent CTF refinement and nonuniform refinement with C4 symmetry imposed, in cryosSPARC.^56^ Outputs from nonuniform refinement underwent cryoSPARC Local Refinement to produce the final 2.85 Å (3.23 Å C1) and 3.98 Å (6.4 Å C1) reconstructions for the Apo and Fructose samples, respectively. Structural biology applications used in this project were compiled and configured by SBGrid.^69^

#### Model building and refinement

A monomeric model of BmG9 was generated using ColabFold.^57^ The rank 1 model was placed in the map with 4-fold symmetry using DockInMap in PHENIX,^58^ and refined through cycles of manual rebuilding in Coot,^59^ real-space refinement in PHENIX with macrocycles including morphing, global minimization, nhq_flips, and ADP, under secondary structure and NCS constraints, and remodeling by simulations run in the ISOLDE plugin of ChimeraX.^60^ The refinement statistics are summarized in Table 1.

#### Electrophysiology

*Xenopus laevis* oocytes (EcoCyte Bioscience) were injected with 20 ng cRNA and cultured at 18°C in ND96 media containing (mM): 2 KCl, 96 NaCl, 2.0 MgCl_2_, 1.8 CaCl_2_, 5 HEPES-NaOH pH 7.4 supplemented with penicillin/streptomycin. Electrophysiological recordings were performed two days after injection by two-electrode voltage clamp using an OC-725 amplifier. Whole-cell currents were elicited by 2.5-s voltage ramp from −150 to +90 mV from a holding potential of −80 mV in ND96 (pH 7.4), filtered at 1 kHz, and recorded in pCLAMP 8 software (Molecular Devices).

#### Generation of multiple sequence alignments

The members of the GR family of proteins display a high degree of functional and sequence diversity which makes their sequence alignment and functional classification a difficult task. To explore the sequence-function relationship of GRs, we first created a seed alignment of 57 different GR sequences from *D. melanogaster* by structurally aligning monomer models downloaded from the AlphaFold Protein Structure Database^26^ using MUSTANG.^61^ Using this seed alignment, we created a profile hidden Markov model (HMM) using the hmmbuild tool from the HMMER software package (hmmer.org) and searched the Uniref. 50 database (release-2023_02) for sequences matching this profile HMM using the hmmsearch tool in HMMER. Using the Uniref. 50 database avoided an overrepresentation of sequences from insect groups with abundant sequence information (like the Drosophilidae). We filtered the set of sequences obtained in the previous step to select sequences with bit score larger than 50, sequence length between 300 and 600, and from species belonging to the class insecta. Next, we added the sequences of known fructose-activated GRs to this filtered set of sequences. We also added sequences of the two odorant receptor proteins with published structures, AbOrco and MhOr5, to generate the final set of 1895 sequences. We then aligned these sequences to the profile HMM created in the first step.

We created a separate multiple sequence alignment of insect ORs using a method similar to the one we used for creating the alignment of GRs described above. We created a profile HMM from structural alignment of 62 models of *D. melanogaster* ORs in the AlphaFold Protein Structure Database^26^ and identified insect sequences of 300–600 amino acids in length. Some of these sequences turned out to be ORCO proteins which we removed from this set, and we added the MhOr5 sequence. Finally, we aligned this set of sequences to the profile HMM that was created in the first step to create an alignment of 3885 OR sequences.

Given the poor annotation of GR and OR sequences, we then investigated whether sequences in our alignments were indeed either GRs or ORs. We randomly selected 10 sequences from either alignment and confirmed by visual inspection that their AlphaFold models showed the stereotypical GR/OR fold. To test whether our two profile HMMs captured distinct features of GRs and ORs, respectively, we attempted to align the BmGr9 sequence to the OR profile HMM and the MhOr5 sequence to the GR profile HMM. Both alignments were unsuccessful, indicating that each profile HMM effectively captures the unique sequence features of its respective protein family.

We used the previously published alignment of 176 ORCO sequences^24^ without any modifications.

#### Phylogenetic tree construction and classification

Using IQ-TREE 2,^49^ we inferred 20 separate maximum likelihood phylogenetic trees from the alignment of 1895 GR sequences with the Le-Gascuel (LG) substitution model,^70^ optimized equilibrium frequencies, and across-site rate variation using the discrete gamma model with 8 categories. We selected the tree with the best log likelihood as the final tree for further analysis. The resulting tree with aBayes posterior probability branch support values is in Figure S10, and the annotated tree in Figure S11. Based on the position of the AbOrco and MhOr5 sequences and annotations of nearby sequences, we determined that one small clade in the tree contained ORs (41 sequences), marking the branchpoint of divergence of ORs from the GR family. These 41 sequences were removed from the ‘all GR’ set of 1854 sequences for further analyses.

For the next part of our analysis, we assumed that GRs that appear in the same subfamily of the tree to be functionally related to each other. This assumption was supported by the fact that all known d-fructose receptors—DmGr43a, BmGr9, AgGr25, Helicoverpa armigera Gr4, and *Apis mellifera* Gr3—clustered in the same subfamily. We extracted the corresponding set of 74 sequences, which we refer to as the Gr43a subfamily. To functionally identify other subfamilies in the tree, we looked for *D. melanogaster* GRs in each subfamily and assigned the function of the *D. melanogaster* GRs found in that subfamily to the entire subfamily. Using this strategy, we extracted two more subfamilies in addition to Gr43a subfamily for further analysis (Figure S11): The Gr63a subfamily containing known CO_2_ receptors (107 sequences) and the Gr5a subfamily containing many known sugar receptors (251 sequences).

#### Sequence conservation analysis

For the OR family and each of the three GR subfamilies chosen for further analysis, we selected a structural model of a representative GR: the agonist-free MhOr5 structure (PDB ID: 7LIC) for the OR family; the agonist-free BmGr9 structure for the Gr43a subfamily; and the AlphaFold2 models of Gr5a (UniProt ID: Q9W497) and Gr63a (UniProt ID: Q9VZL7) for the Gr5a and Gr63a subfamilies, respectively. For each of the five corresponding sequences alignments, we performed sequence conservation analysis using ConSurf,^32^ using the WAG amino acid substitution model.

#### Sequence covariation analysis

Using the EVcouplings python framework,^48^ we performed sequence covariation analysis on each alignment (GRs and ORs). Couplings were inferred using the pseudolikelihood maximization and coupling scores and probabilities were calculated using logistic regression. We used an 8 Å cutoff for the Cα-to-Cα distance as suggested in the EVcouplings tutorial to create the intrasubunit contact maps of BmGr9 and MhOr5. We projected the top evolutionarily coupled pairs with probabilities greater than 90% for each analysis on the respective contact map and structure, from the GRs alignment on BmGr9 and from the ORs alignment on MhOr5.

#### Ligand-binding pocket analysis

The predicted ligand-binding pockets for the Gr5a and Gr63a subfamilies were identified in the central region of the transmembrane S1-S6 bundle of each representative AlphaFold2 model. We confirmed that the approximate size and location of each predicted pocket was a reasonable representative by comparing them to 4–5 additional AlphaFold2 models chosen from different branches of the same subfamily. The experimentally determined ligand-binding pocket of BmGr9 was used for the Gr43a subfamily and of MhOr5 for the OR family. All residue positions with solvent-exposed atoms facing the respective pockets were selected (Figure S12). The observed amino acid frequencies at each position were computed from the respective sequence alignment. The heatmaps in Figure 6 were obtained by summing the amino acid frequencies according to the following amino acid groupings based on chemical properties: aliphatic (Ala, Cys, Leu, Met, Val; black), polar (Asn, Gln, Ser, Thr; orange), negatively charged (Asp, Glu; red), positively charged (Arg, Lys; blue), aromatic (His, Phe, Trp, Tyr; green), and shape-determining (Gly, Pro; pink) residues.

#### Determination of loop lengths

Despite high diversity and low sequence similarity of GR proteins, our structure-based and profile HMM-assisted approach produced an alignment with high overlap (>80%) coverage for the transmembrane helix regions (Figure S6B). Assuming that sequence regions corresponding to transmembrane helices are well aligned with each other, the poorly aligned regions between two transmembrane helices must correspond to the loops that connect those helices. Because we have the experimentally determined structure of BmGr9, we used its sequence as a reference to define boundaries of helices S1 to S7 for all sequences in the GR alignment and the selected subfamily alignments (helix boundaries for S1 are residues 52–79; S2, 90–135; S3, 141–176; S4, 178–229; S5, 287–339; S6, 345–389; S7a, 395–409; S7b, 424–444). For the OR alignment, we similarly used the MhOr5 structure as a reference (S1, 49–76; S2, 78–123; S3, 130–159; S4, 198–251; S5, 317–368; S6, 371–415; S7a, 420–434; S7b, 448–472). To determine the loop lengths, we counted the number of residues connecting the two adjacent transmembrane helices in each sequence, after shortening each helix by 1 residue on each end to allow for noise in the alignment. To avoid including incomplete sequences, we only counted sequences that had non-zero loop length.

#### Pore dimension analysis

We used the HOLE program^62^ to analyze the dimensions of ion conduction pores of agonist-free and fructose-bound structures of BmGr9.

#### Fructose docking

We used AutoDock Vina^63,64^ to dock β-_D_-fructopyranose in the fructose-bound structure. The AutoDock search space was set to a cuboid of 25 × 30 × 40 Å^3^ spanning the length and width of a monomer. We then used real-space refinement in PHENIX^58^ (macrocycles including morphing, global minimization, nhq_flips, and ADP, under secondary structure and NCS constraints) with the AutoDock rank 1 position of fructose which modeled the fructose into the density.

#### Molecular modeling of simulation systems

To set up the molecular dynamics simulations systems using VMD^46^ (version 1.9.3), we first added hydrogens to the agonist-free BmGr9 structure, and we used PROPKA (version 3.0)^65^ to determine amino acid protonation states at pH 7.0. To account for the missing intracellular S4-S5 loop, we split each BmGr9 subunit into two protein chains resulting in eight separate chains. We neutralized the N and C termini of each chain by patching them with acetyl and N-methylamide groups, respectively. Next, we created three different simulation systems from this model of BmGr9. In the first system, we modeled the pore-penetrating lipids as 1-palmitoyl2-oleoyl-*sn*-glycero-3-phosphatidylcholine (POPC) molecules, while in the second system we modeled these lipids as 1-palmitoyl-2-oleoyl-*sn*-glycero-3-phosphatidylethanolamine (POPE). In the third system, we completely removed the pore-penetrating lipids. Next we used CHARMM-GUI^67^ to embed each of these molecular models in mixed lipid membranes composed of 55% POPE, 19% POPC, 14% 1-palmitoyl-2-oleoyl-*sn*-glycero-3-phoshatidylinositol (POPI), 6% 1-palmitoyl-2-oleoyl-*sn*-glycero-3-phosphatidylserine (POPS), and 6% cholesterol to approximate the membrane composition of insect cells.^28–30^ We hydrated each system with TIP3P explicit water and neutralized them with KCl to a final concentration of 0.15 M. Each of the three different simulation systems contains a total of ~200,000 atoms.

#### Molecular dynamics simulations

We performed molecular dynamics simulations on all three simulation systems at 298 K and 1 atm pressure using Langevin thermostat and hybrid Nosé-Hoover pressure control in the *NpT* ensemble using the following protocol. In the first step, we constrained all atoms to their initial positions except the lipid tails, which we minimized for 1000 steps and simulated for 0.5 ns. In the next step, we hydrated the pore by minimizing water and all lipids except the headgroups of the pore-penetrating lipids for 5000 steps and simulating for 10 ns. Following this, we relaxed the system by minimizing protein sidechains and the pore-penetrating lipids for another 5000 steps and simulating for 10 ns. Finally, we performed free dynamics of the entire system for another 10 ns. During these initial 4 steps, Langevin damping coefficient was set to 1.0 ps^−1^. Following this, we performed a 200-ns production for each system with Langevin damping coefficient set to 0.1 ps^−1^.

We used GPU NAMD 2.14^47^ and CHARMM36m forcefield^71^ with a timestep of 2 fs for all simulations. We calculated nonbonded interactions using a switching function with 12 Å cutoff and a switch distance of 10 Å and used the particle mesh Ewald (PME) method with periodic boundary conditions and a grid density of greater than 1 Å^−3^ to calculate electrostatic interactions. In all simulations, we constrained the N and C termini of the two chains within each subunit at a distance identical to the distance in the initial structure using weak harmonic restraints.

#### Data presentation

We used PyMOL (Schrödinger LLC) to render molecular scenes and interactive Tree of Life (iTOL)^50^ to render phylogenetic trees. The diagram in Figure 3E was made with LigPlot+.^66^ Sequence logos were generated with WebLogo^68^ and colored according to amino acid chemical properties: green, polar amino acids (G, S, T, Y, C, Q, N); blue, basic (K, R, H); red, acidic (D, E); and black, hydrophobic (A, V, L, I, P, W, F, M). We used matplotlib and bioviper packages in python to make the alignment coverage plots and used xmgrace to make protein RMSD plots.

### QUANTIFICATION AND STATISTICAL ANALYSIS

For the electrophysiology experiments described in Figure 1A, S1A–S1D, n refers to independent biological replicates. Shapiro-Wilk tests were used to assess the normality of all datasets (p ≤ 0.05 rejected normal distribution). Nonparametric tests consisted of Kruskal-Wallis followed by a Steel-Dwass post hoc test for multiple comparisons (JMP11, SAS).

## Supplementary Material

1

## Figures and Tables

**Table 1.** Cryo-EM data collection, refinement, and validation statistics

	Agonist-free BmGR9	Fructose-bound BmGR9

Cryo-EM map	EMD-43129	EMD-43130
PDB	8VC1	8VC2

Data collection and processing		

Magnification	60,240	60,240
Voltage (kV)	300	300
Electron exposure (e^−^/Å^−2^)	74.492	80.144
Defocus range (mm)	0.8, 2.2	0.8, 2.2
Pixel size (Å)	0.83	0.83
Symmetry imposed	C4	C4
Initial particle images (no.)	1,955,549	1,141,024
Final particle images (no.)	1,011,451	233,351
Map resolution (Å)	2.85	3.98
FSC threshold	0.143	0.143
Map resolution range (Å)	2.51–9.3	3.4–10.9

Refinement		

Initial model used	ColabFold-generated	agonist-free BmGr9
Model resolution (Å)	3.00	4.20
FSC threshold	0.500	0.500
Map-sharpening B-factor (Å^2^)	150.9	181.3
Model composition		
Non-hydrogen atoms	13,128	11,796
Protein residues	1,564	1,516
B factors (Å^2^)		
Protein	58.19	43.02
RMSD		
Bond lengths (Å)	0.01	0.003
Bond angles (°)	0.977	0.709

Validation		

MolProbity score	1.20	1.16
Clashscore	4.03	2.91
Poor rotamers (%)	0.30	0.31
Ramachandran plot		
Favored (%)	97.83	97.60
Allowed (%)	2.07	2.40
Disallowed (%)	0.00	0.00

EMD, Electron Microscopy Database; PDB, Protein Data Bank; FSC, Fourier shell correlation; RMSD, root-mean-square deviation.		

**KEY RESOURCES TABLE:** 

REAGENT or RESOURCE	SOURCE	IDENTIFIER

Bacterial and virus strains		

*E. coli* DH5α	Thermo Fisher Scientific	EC0112

Biological samples		

*Xenopus laevis* oocytes	EcoCyte Bioscience	N/A

Chemicals, peptides, and recombinant proteins		

n-dodecyl β-D-maltoside (DDM)	Anatrace	D310S
glyco-diosgenin (GDN)	Anatrace	GDN101

Deposited data		

BmGr9 agonist-free structure	This paper	PDB: 8VC1
BmGr9 fructose-bound structure	This paper	PDB: 8VC2
BmGr9 agonist-free cryo-EM map	This paper	EMD-43129
BmGr9 fructose-bound cryo-EM map	This paper	EMD-43130
GR family sequence alignment and phylogenetic tree, OR family sequence alignment, and molecular dynamics trajectories of BmGr9	This paper	https://doi.org/10.5061/dryad.cc2fqz6dp

Experimental models: Cell lines		

HEK293T	ATCC	CRL-3216
HEK293F inducible GnTI- suspension cells	Thermo Fisher Scientific	A39242

Oligonucleotides		

Double-stranded DNA to insert N-terminal Twin-Strep tag in pHR-CMV-TetO2_3C-Twin-Strep: AGTGAACC GTCTGATCTCAACAAGCTGTCTAGAGCCACCATGG GTTGGAGCCATCCACAGTTCGAAAAAGGTGGAGGT TCTGGCGGTGGATCAGGTGGAAGTGCATGGTCTCA CCCTCAGTTTGAGAAAGGAGGTAGTGGATCTGCTGA ATTCGTGAGCAAGGGCGAGGAGCTGTTCACC	IDT	N/A
Forward primer to insert BmGr9 into modified pHR-CMV-TetO2_3C-Twin-Strep: CTGTTTCAGGGACCAGGTACCATGCCTCCT TCGCCAGATCTG	IDT	N/A
Reverse primer to insert BmGr9 into modified pHR- CMV-TetO2_3C-Twin-Strep: GGAGGGAGAGGGG CGCTCGAGTCATTAACTATCATATCGCTGGAA	IDT	N/A
Forward primer to insert N-terminal EmGFP into pHR-CMV-TetO2_EmGFP: ATGGTGAGCAAGGG CGAGGAG	IDT	N/A
Reverse primer to insert N-terminal EmGFP into pHR-CMV-TetO2_EmGFP: TGCGGCCGCTGGT CCCTGAAACAGCACCTCAAGTGCTGCCTTGTA CAGCTCGTCCATGC	IDT	N/A
Forward primer to amplify pHR-CMV-TetO2_EmGFP: GCAGCACTTGAGGTGCTGTTTCAGGGACCAGCGG CCGCATAATGACTCGAGGACTCTTG	IDT	N/A
Reverse primer to amplify pHR-CMV-TetO2_EmGFP: CTCCTCGCCCTTGCTCACCAT	IDT	N/A
Forward primer to insert BmGr9 into modified pHR- CMV-TetO2_EmGFP: TTTCAGGGACCAGCGGCCGCAATGCCTCCTTCG CCAGATCTG	IDT	N/A
Reverse primer to insert BmGr9 into modified pHR-CMV-TetO2_EmGFP: CTCGACTCAAGAGTCCTCGAGTTAACTATCATAT CGCTGGAA	IDT	N/A

Recombinant DNA		

pHR-CMV-TetO2_3C-Twin-Strep	Elegheert et al.^45^	Addgene ID: 113883
pOX	N/A	Addgene ID: 3780
pHR-CMV-TetO2_EmGFP	Elegheert et al.^45^	Addgene ID: 113892
psPAX2 packaging plasmid	Elegheert et al.^45^	Addgene ID: 12260
pMD2.G envelope plasmid	Elegheert et al.^45^	Addgene ID: 12259
Plasmid: Twin-Strep-tagged-BmGr9 in pHR-CMV-TetO2_3C-Twin-Strep	This paper	N/A
Plasmid: Twin-Strep-tagged-BmGr9 in pOX	This paper	N/A
Plasmid: EmGFP-tagged-BmGr9 in pHR-CMV- TetO2_EmGFP	This paper	N/A

Software and algorithms		

Visual Molecular Dynamics (VMD) 1.9.3	Humphrey et al.^46^	https://www.ks.uiuc.edu/Research/vmd/vmd-1.9.3/ RRID: SCR_001820
NAMD 2.14 CUDA	Phillips et al.^47^	https://www.ks.uiuc.edu/Research/namd/ RRID: SCR_014894
Python version 2.10	Python Software Foundation	https://www.python.org/ RRID: SCR_008394
EVCouplings version 0.1.2	Hopf et al.^48^	https://github.com/debbiemarkslab/EVcouplings RRID: SCR_018745
PyMOL 2.5	Schrö dinger LLC	https://pymol.org/2/ RRID :SCR_000305
IQ-TREE multicore 2.1.2	Minh et al.^49^	http://www.iqtree.org/ RRID:SCR_017254
Bioviper 0.1.2	Samuel P. Berry (Gaudet Lab)	https://github.com/samberry19/bioviper
Interactive Tree of Life (iTOL) version 6	Letunic et al.^50^	https://itol.embl.de/ RRID: SCR_018174
xmgrace	Plasma Laboratory	https://plasma-gate.weizmann.ac.il/Grace/
SerialEM version 4.1	Mastronarde et al.^51^	https://bio3d.colorado.edu/SerialEM/ RRID: SCR_017293
MotionCor2 version 1.6.4	Zheng et al.^52^	https://emcore.ucsf.edu/ucsf-software RRID: SCR_016499
CTFFIND4 version 4.1.14	Rohou et al.^53^	https://grigoriefflab.umassmed.edu/ctf_estimation_ctffind_ctftilt RRID: SCR_016732
crYOLO version 1.9.6	Wagner et al.^54^	http://sphire.mpg.de/wiki/doku.php?id=pipeline:window:cryolo RRID: SCR_018392
RELION version 4.0.1	Scheres et al.^55^	http://www2.mrc-lmb.cam.ac.uk/relion RRID: SCR_016274
cryoSPARC version 3.3.2	Punjani et al.^56^	https://cryosparc.com RRID: SCR_016501
ColabFold 1.5.2	Mirdita et al.^57^	https://colab.research.google.com/github/sokrypton/ColabFold/blob/main/AlphaFold2.ipynb
PHENIX version 1.21–4487	Liebschner et al.^58^	https://phenix-online.org RRID: SCR_014224
Coot version 0.9.8.91	Emsley et al.^59^	https://www2.mrc-lmb.cam.ac.uk/personal/pemsley/coot/ RRID: SCR_014222
ISOLDE/ChimeraX version 1.6.1	Croll et al.^60^	https://tristanic.github.io/isolde/ RRID: SCR_015872
HMMER 3.2.1	Sean Eddy Lab	hmmer.org RRID: SCR_005305
MUSTANG version 3.2.4	Konagurthu et al.^61^	https://lcb.infotech.monash.edu/mustang/
AlphaFold2 structure database	Tunyasuvunakool et al.^26^	https://alphafold.ebi.ac.uk/faq
Consurf	Ashkenazy et al.^32^	https://consurfdb.tau.ac.il/
Hole version 2.2.005	Smart et al.^62^	https://www.holeprogram.org/
AutoDock Vina version 1.1.2	Trott et al.^63^ Eberhardt et al.^64^	https://vina.scripps.edu/ RRID: SCR_011958
PROPKA 3.0	Olsson et al.^65^	https://server.poissonboltzmann.org/pdb2pqr
LigPlot+ version 2.2.8	Laskowski et al.^66^	https://www.ebi.ac.uk/thornton-srv/software/LigPlus/ RRID: SCR_018249
CHARMM-GUI version 3.8	Jo et al.^67^	https://www.charmm-gui.org/
WebLogo 3	Crooks et al.^68^	https://weblogo.threeplusone.com/create.cgi RRID: SCR_010236

Other		

400 mesh Quantifoil Cu 1.2/1.3 grids	Electron Microscopy Sciences	Q450CR1.3

## Highlights

- Agonist-free and fructose-bound structures of *Bombyx mori* gustatory receptor Gr9
- BmGr9 resembles odorant receptors, with a larger, solvent-accessible ligand pocket
- Pore-penetrating lipids inhabit intersubunit fenestrations of both BmGr9 and ORs
- Distinct GR families show different patterns of ligand pocket residue conservation
